# The Association of Health Literacy with Illness and Medication Beliefs among Patients with Chronic Obstructive Pulmonary Disease

**DOI:** 10.1371/journal.pone.0123937

**Published:** 2015-04-27

**Authors:** Minal S. Kale, Alex D. Federman, Katherine Krauskopf, Michael Wolf, Rachel O’Conor, Melissa Martynenko, Howard Leventhal, Juan P. Wisnivesky

**Affiliations:** 1 Division of General Internal Medicine, Icahn School of Medicine at Mount Sinai, New York, New York, United States of America; 2 Division of General Internal Medicine, Feinberg School of Medicine, Northwestern University, Chicago, Illinois, United States of America; 3 Institute for Health, Health Care Policy and Aging Research, Rutgers, The State University of New Jersey, New Brunswick, New Jersey, United States of America; 4 Division of Pulmonary, Critical Care and Sleep Medicine, Icahn School of Medicine at Mount Sinai, New York, New York, United States of America; Sickle Cell Unit, JAMAICA

## Abstract

**Background:**

Low health literacy is associated with low adherence to self-management in many chronic diseases. Additionally, health beliefs are thought to be determinants of self-management behaviors. In this study we sought to determine the association, if any, of health literacy and health beliefs among elderly individuals with COPD.

**Methods:**

We enrolled a cohort of patients with COPD from two academic urban settings in New York, NY and Chicago, IL. Health literacy was measured using the Short Test of Functional Health Literacy in Adults. Using the framework of the Self-Regulation Model, illness and medication beliefs were measured with the Brief Illness Perception Questionnaire (B-IPQ) and Beliefs about Medications Questionnaire (BMQ). Unadjusted analyses, with corresponding Cohen’s d effect sizes, and multiple logistic regression were used to assess the relationships between HL and illness and medication beliefs.

**Results:**

We enrolled 235 participants, 29% of whom had low health literacy. Patients with low health literacy were more likely to belong to a racial minority group (p<0.001), not be married (p = 0.006), and to have lower income (p<0.001) or education (p<0.001). In unadjusted analyses, patients with low health literacy were less likely to believe they will always have COPD (p = 0.003, Cohen’s *d* = 0.42), and were more likely to be concerned about their illness ((p = 0.04, Cohen’s d = 0.17). In analyses adjusted for sociodemographic factors and other health beliefs, patients with low health literacy were less likely to believe that they will always have COPD (odds ratio [OR]: 0.78, 95% confidence interval [CI]: 0.65–0.94). In addition, the association of low health literacy with expressed concern about medications remained significant (OR: 1.20, 95% CI: 1.05–1.37) though the association of low health literacy with belief in the necessity of medications was no longer significant (OR: 0.92, 95% CI: 0.82–1.04).

**Conclusions:**

In this cohort of urban individuals with COPD, low health literacy was prevalent, and associated with illness beliefs that predict decreased adherence. Our results suggest that targeted strategies to address low health literacy and related illness and medications beliefs might improve COPD medication adherence and other self-management behaviors.

## Introduction

Chronic obstructive pulmonary disease (COPD) is a common medical condition associated with substantial morbidity, mortality, and high resource utilization[1–3]. Although COPD is a progressive illness, disease self-management, such as adherence to chronic daily medications, can reduce the frequency of exacerbations and hospitalizations and may impact mortality [4]. Unfortunately, several studies have demonstrated that up to half of patients with COPD do not adhere to medication or perform other important self-management behaviors, such as effective inhaler use [5–11].

There is a growing body of research suggesting that health literacy is an important determinant of self-management behaviors and outcomes in a variety of chronic diseases [12–14]. Health literacy is defined as the ability to obtain, process, and understand basic health information [15]. Low health literacy can negatively impact disease outcomes by compromising patient-provider communication about medications, limiting patients’ understanding of the treatment rationale, and interfering with the ability to acquire medications, among others. Self-management behaviors that are potentially impacted not only include medication adherence, but also proper inhaler technique, the appropriate use of action plans, guideline-concordant vaccination administration, as well as routine COPD care. However, the pathways underlying the relationship between low health literacy and self-management behaviors have not been clearly established. This gap in knowledge constitutes a barrier to designing interventions to address COPD self-management in low-literacy patients.

Current behavioral models emphasize the role of health beliefs in patient decision-making about disease self-management [16–19]. Cognitive illness representations, emotional responses to the disease, as well as medication beliefs have been shown to be associated with adherence to self-management behaviors in COPD and other chronic diseases [16–19]. An association between health beliefs and health literacy may be expected as the process for establishing a mental model of chronic diseases such as COPD is likely contingent on information-seeking and processing.

Despite the relatively high prevalence of low health literacy among individuals with COPD, its relationship to health beliefs is not well understood. Understanding which specific beliefs are frequently held by low health literate COPD patients is a critical component of delivering behaviorally focused education and counseling. In this study, we examine the association of health beliefs with health literacy in a prospective cohort of COPD patients, hypothesizing that low health literacy is associated with a mental model of disease that may negatively impact COPD self-management.

## Methods

### Study Participants

The study was conducted using data collected from COPD patients enrolled into a prospective, longitudinal, multi-site observational cohort study. The overall objective of this study is to investigate longitudinal associations between cognition, health literacy, and self-care in individuals with COPD. Participants were recruited from three socioeconomically and racially diverse inner-city outpatient clinics in New York City, NY and Chicago, IL between December 2011 and June 2013. The study was approved by the Institutional Review Boards of the Icahn School of Medicine at Mount Sinai and the Northwestern University Feinberg School of Medicine.

Patients eligible for the study were ≥55 years of age, speakers of English or Spanish, community-dwelling, and carried a physician’s diagnosis of COPD. Exclusion criteria included a history of dementia and other neurological and psychological conditions profoundly affecting cognition (for example, stroke, Parkinson’s disease, and schizophrenia) as documented in the electronic medical record (EMR) with International Classification of Diseases, 9^th^ Revision codes. Potential participants were identified from reviews of the EMR and clinic registration systems of participating clinics. Trained, bilingual research assistants contacted patients by telephone, and conducted a brief eligibility screener. If eligible, patients were scheduled for an in-person interview in English or Spanish, at which time formal consent was obtained. All participants were asked to read and sign a written informed consent form and HIPAA form in either English or Spanish. The consent form was approved by the Institutional Review Boards of the Icahn School of Medicine at Mount Sinai and the Northwestern University Feinberg School of Medicine.

### Health Literacy

Health literacy was assessed with the Short Test of Functional Health Literacy in Adults (S-TOFHLA)[20–21]. The S-TOFHLA is composed of a 36-item reading comprehension section and a 4-item numeracy exercise. The instrument is valid, reliable, strongly correlated with intermediate and long-term health outcomes, and is available in English and Spanish [20–21]. The reading comprehension section is presented as two timed (7 minutes), clinically oriented reading passages that omit key words and phrases from every line. The total score range is 0–36; scores were classified as low (<23) according to a well-validated cut-point [21].

### COPD Beliefs

The study was grounded in the Self-Regulation Model (SRM)[22]. The SRM posits that patients develop cognitive representations of chronic diseases based on prior health experiences. These illness representations, in combination with emotional responses to symptoms, activate behavioral actions and guide coping procedures. The SRM defines five domains of illness representations: identity, timeline, cause, consequences, and control. Identity refers to the patient’s identification of symptoms, including whether patients attribute their cough, shortness of breath, or exercise limitation to COPD, other conditions, or aging. Timeline incorporates beliefs about the chronicity of COPD and natural progression. According to the model, patients who believe that COPD is a progressive disease will be more likely to adhere to recommended self-management behaviors. Cause refers to personal ideas about illness etiology, for example, that COPD is due to smoking, stress, lifestyle, genetic factors, or others. Control encompasses the beliefs and expectations that COPD symptoms can be controlled, either by personal action or treatment. Consequences refer to the expected impact of COPD on health and functional status. The SRM predicts that patients who minimize the potential consequences of the disease may be less likely to attempt quitting smoking or to follow other physician recommendations. Emotional representations, such as fear, anger, anxiety, or sadness activated by the disease or its symptoms may also influence patients’ self-management behaviors and heath care seeking according to the model.

Patients’ mental models, i.e. representations, of COPD were assessed using the Brief-Illness Perceptions Questionnaire, a validated, 10-question instrument available in English and Spanish [23–25]. The B-IPQ includes items assessing beliefs along the 5 cognitive domains of the SRM as well as emotional responses to COPD. Each item is assessed on a scale of 0–10 with greater scores indicating greater disease congruence (i.e., a mental model consistent with COPD as a serious, progressive disease).

### Medication Beliefs

Cognitive representations of treatment can also influence self-management. In the Necessity-Concerns Framework, adherence to treatment is considered the result of an interplay between patients’ beliefs in their need for treatment and their concerns about potential adverse consequences, such as toxicities and dependence. Patients who report high concerns and low necessity beliefs are more likely to be non-adherent. In order to assess medication beliefs, we used Horne’s Beliefs in Medicines Questionnaire (BMQ). The BMQ consists of two 5-item subscales representing beliefs about medication necessity and concerns about harmful effects. The scale is available in English and Spanish and has been used in mixed populations with chronic diseases [26]. The responses within each sub-domain are summed for a total score ranging from 5–25, with higher scores indicating more negative beliefs.

### Sociodemographic Characteristics and other Covariates

Age, sex, race, ethnicity, income, education, general health, and access to a usual source of care for COPD were assessed by self-report using validated items [27]. Severity of COPD was gauged with the COPD severity index, a validated self-report measure that uses information on respiratory symptoms, use of systemic corticosteroids and other COPD medications, previous hospitalization for COPD, history of mechanical ventilation, and home oxygen use [28]. The index correlates well with physiologic measures of COPD severity and health-related quality of life; scores range from 0–25 with higher values indicating increased severity. Pulmonary function was measured by spirometry and patients were categorized according to the Global Initiative for Chronic Obstructive Lung Disease (GOLD) [29]. The presence of comorbidities including, coronary artery disease (CAD), congestive heart failure (CHF), and diabetes mellitus were collected by self-report and confirmed through chart abstraction.

### Statistical Analysis

We assessed differences in baseline characteristics of COPD patients with low and adequate health literacy using the chi-square or Fisher’s exact test, as appropriate. We then evaluated the unadjusted associations between illness and medication beliefs with health literacy using the Wilcoxon rank sum test and measured the effect size with Cohen’s D statistic. Consistent with the literature, we used established cutoffs of 0.2, 0.5, and 0.8 for a small, moderate, and large effect size respectively [30]. Logistic regression analysis was used to test the association of health beliefs with low-health literacy after adjusting for sociodemographic characteristics and the presence of a regular physician to manage COPD. All analyses were performed using SAS statistical software (SAS Institute, Cary, NC), using two-sided *p*-values.

## Results

We screened 591 potential participants identified by electronic searches; of these, 74 were ineligible, 5 passively declined, 172 refused, and 100 expressed interest but had not been enrolled at the time of this analysis. Of the eligible 240 participants, 235 completed the baseline questionnaire. Overall, 66 (29%) COPD patients had STOFHLA scores <23 indicating low health literacy (Table 1). There were no significant differences in the age or sex distribution between patients with low and adequate health literacy (p>0.05 for both comparisons). Patients with low health literacy were more likely to belong to a racial minority group (p<0.001), not be married (p = 0.006), and to have lower income (p<0.001) or education (p<0.001). General health, COPD severity, and pulmonary function according to GOLD categories were not significantly different between the two groups (p>0.05 for all comparisons). Diabetes mellitus was more common among low health literate patients (p<0.001), there were no significant differences in presence of CAD or CHF. Low health literacy was also associated with lack of a usual source of care for COPD (p = 0.004).

**Table 1:** Baseline Characteristics by Health Literacy Status.

Characteristic		Total(n = 235)	Health Literacy		P-value
		n (%)	Low (n = 66)	Adequate (n = 169)	
			n (%)	n (%)	
**Age**					
	55–64	82 (36)	19 (29)	63 (38)	0.35
	65–70	51 (22)	15 (23)	36 (22)	
	70–74	97 (42)	32 (48)	65 (40)	
**Female**		150 (64)	43 (65)	107 (63)	0.79
**Married**		70 (30)	11 (17)	59 (35)	0.006
**Race**					
	White	89 (38)	10 (15	79 (47)	<0.001
	Black	107 (46)	36 (55)	71 (43)	
	Hispanic	33 (14)	17 (26)	16 (10)	
	Other	5 (2)	3 (5)	2 (1)	
**Monthly Income**					
	$0-$750	42 (19)	20 (31)	22 (14)	<0.001
	$751-$1350	67 (30)	27 (42)	40 (25)	
	$1351-$3000	61 (27)	16 (25)	45 (28)	
	**>**$3000	57 (25)	1 (2)	56 (34)	
**Education**					
	Less than 12 years	54 (23)	36 (55)	18 (11)	<0.001
	High school graduate	47 (20)	11 (17)	36 (21)	
	Some college	60 (26)	11 (17)	49 (29)	
	College degree, or higher	73 (31)	7 (11)	66 (39)	
**General health**					
	Excellent/very good	53 (23)	10 (15)	43 (25)	0.07
	Good	87 (37)	22 (33)	65 (38)	
	Fair/poor	95 (40)	34 (52)	61 (36)	
**Comorbidities**					
	CAD	32 (14)	12 (18)	20 (12)	0.21
	CHF	28 (12)	12 (18)	16 (10)	0.07
	DM	58 (25)	27 (41)	31 (18)	<0.001
**COPD Severity, mean (STD)**		5.5 (3.7)	6.3 (4.0)	5.5 (3.7)	0.18
**Pulmonary Function (GOLD)**					
	Mild	62 (28)	18 (28)	44 (28)	0.62
	Moderate	104 (46)	26 (41)	78 (49)	
	Severe	48 (21)	17 (27)	31 (19)	
	Very severe	10 (4)	3 (5)	7 (4)	
**Usual source of care**		190 (81)	45 (69)	145 (86)	0.004

Differences between patients with low and adequate health literacy were statistically significant in one of the 5 components of the representations of COPD and both of the factors involving medications (Table 2). Among cognitive illness representations, patients with low health literacy were less likely to believe that they will always have COPD (timeline domain; p = 0.003, Cohen’s *d* = 0.42). However, patients with low and adequate health literacy were equally likely to experience symptoms of COPD (identity domain; p = 0.50, Cohen’s *d* = 0.07), believe in their personal control of the disease (control domain; p = 0.06, Cohen’s *d* = 0.21), believe in treatment control of COPD (control domain; p = 0.58, Cohen’s *d* = 0.04), and to report that COPD affected their lives (consequences domain; p = 0.27, Cohen’s *d* = 0.15). Among the emotional representations, COPD patients with low health literacy were more likely to be concerned about their illness (p = 0.04, Cohen’s *d* = 0.17) and to report that their COPD affected their emotions (p = 0.05, Cohen’s *d* = 0.25). While patients with low health literacy were more likely to believe that COPD medications were necessary (p = 0.005, Cohen’s *d* = 0.48), they were also more likely to express concerns about the potential negative consequences of their medications (p<0.001, Cohen’s *d* = 0.55).

**Table 2:** Unadjusted Associations of Low Health Literacy with Health and COPD Controller Medication Beliefs.

Cognitive illness representations	Low	Adequate	P-value	Cohen’s D
	N = 66	N = 169		
Affects life, mean (sd)	6.2 (3.1)	5.8 (2.7)	0.27	0.149
Will always have COPD, mean (sd)	8.0 (3.0)	9.1 (2.2)	0.003	0.418
Personal control of COPD, mean (sd)	6.9 (3.1)	6.3 (2.5)	0.06	0.213
Treatment control of COPD, mean (sd)	7.6 (2.6)	7.5 (2.6)	0.58	0.038
Experience symptoms, mean (sd)	6.2 (3.2)	6.0 (2.7)	0.50	0.068
**Emotional representations**				
Concern about COPD, mean (sd)	8.6 (2.7)	8.0 (2.9)	0.04	0.169
COPD affects emotions, mean (sd)	6.3 (3.7)	5.4 (3.4)	0.05	0.253
**Illness comprehension**				
Understands COPD, mean (sd)	8.0 (2.7)	8.3 (2.3)	0.94	0.120
**Medication beliefs**				
Medications not necessary, mean (sd)	13.0 (3.8)	15.0 (4.5)	0.005	0.480
Concerned about medications, mean (sd)	14.7 (3.7)	12.6 (4.0)	<0.001	0.545

Controlling for sex, age, marital status, race, education, having a usual source of care, and COPD severity (Table 3) did not remove the association of health literacy with the belief that COPD is a life-long illness; patients scoring low in health literacy remained less likely to believe they will always have COPD (odds ratio [OR]: 0.78, 95% confidence interval [CI]: 0.65–0.94). In addition, the association of low health literacy with expressed concern about medications remained significant (OR: 1.20, 95% CI: 1.05–1.37) though the association of low health literacy with belief in the necessity of medications was no longer significant (OR: 0.92, 95% CI: 0.82–1.04). The adjustment for the full set of moderating factors eliminated the association of low health literacy with both emotional factors, i.e., concern about COPD (OR: 1.11, 95% CI: 0.92–1.35) and the belief that COPD affects emotions (OR: 0.96, 95% CI: 0.83–1.11).

**Table 3:** Adjusted Associations of Low Health Literacy with Health and COPD Controller Medication Beliefs.

Beliefs*	Adjusted OR
	(95% CI)
Will always have COPD	0.73 (0.60–0.89)
Concern about COPD	1.20 (0.94–1.53)
COPD affects emotions	0.88 (0.75–1.04)
Medications not necessary	0.94 (0.83–1.05)
Concerned about medications	1.15 (1.00–1.31)

*Adjusted model: Low health literacy = beliefs + sex + age + married + race + education + having an MD + COPD severity

## Discussion

In this multicenter cohort study of COPD patients, we found that low health literacy was independently associated with several health beliefs that are important determinants of adherence to self-management behaviors. Specifically, individuals with low health literacy were less likely to believe in the chronicity of their illness and more likely to have greater negative emotional representations of their disease, including greater concern about their illness. Our findings contribute to an emergent literature on the pathways that may underlie the association of health literacy with self-management behaviors in chronic illness. Additionally, we identified potential targets in COPD patients with low health literacy that may be used to improve patient-provider communication. By addressing key disease beliefs, not only will patient understanding of their disease increase, but it may also lead to improved patient adherence to self-management behaviors.

There is strong evidence of the association of health literacy and health outcomes [31]. In one study of 3260 elderly Medicare enrollees, inadequate health literacy was associated with higher mortality rates after controlling for cognitive function, physical functioning, and health lifestyle [12]. Similarly, several studies have demonstrated that individuals with low health literacy are less likely to obtain preventive services such as mammography screening [32–34]. Although there is a paucity of studies examining health literacy in the COPD population specifically, one systematic assessment found an association of low health literacy and greater COPD severity, COPD helplessness, and worse respiratory-specific health-related quality of life [35]. Thus, addressing the mechanisms and impact of health literacy on COPD may translate into improved outcomes in this vulnerable population.

The pathway by which health literacy is associated with worse disease outcomes is not well established. One existing conceptual model postulates that health literacy, among other factors, influences health beliefs and self-care management, which in turn affects disease outcomes [36]. In previous work investigating beliefs and self-management behaviors in older patients with asthma, a prevalent and chronic respiratory disease which also requires daily use of inhaled medications, inaccurate health beliefs about asthma symptoms and treatments were associated with poor adherence [37]. Similarly, in a cohort study of 151 urban diabetics, 28% of whom had low adherence, illness and medication beliefs that were related to the time course of the disease were found to highly influence medication adherence [38]. In the few existing studies in patients with COPD, investigators have found that increased medication concerns [39] and confusion about illness management [7] were associated with low adherence. Through these studies and others, health beliefs are increasingly recognized as an important determinant of COPD self-care [40].

There is a growing understanding of the relationship between health literacy and health beliefs. In one study among patients with HIV, low health literacy was associated with erroneous beliefs regarding HIV transmission and treatment [41]. Additionally, in a study of health literacy and health beliefs in individuals with asthma, low health literacy was associated with several asthma-related beliefs that have been shown to predict poor adherence, such as a decreased belief in the chronicity of asthma [42]. Our finding that there is an association of low health literacy and a decreased belief in the chronicity of COPD may be partially explained by the nature of the disease experience. COPD is a symptomatic illness characterized by the presence of chronic respiratory symptoms ranging from minor to severe, such as chronic cough, fatigue, and dyspnea on exertion. These chronic symptoms may become the new normal, except during acute flares. This disease experience has the potential to translate into the belief that COPD is an acute, episodic disease, and only present during attacks. Addressing this health belief among low health literate patients represents an opportunity to remove a barrier to adherence to chronic daily medications, a mainstay of treatment for COPD.

Limitations to our study must be noted. Although we enrolled a relatively large cohort of inner-city adults with predominant representation of racial minorities, it is possible that our results may not be generalizable to non-urban communities. However, given the high risk of poor outcomes among minority COPD patients, our attention to this population is warranted. It is also possible that our sample may under-represent the true proportion of low health literacy in the sampled population, as health literacy level may have influenced the decision to participate in the study. However, our study refusal rate is consistent with those of other observational studies in similar populations. In addition, our cohort somewhat over-represents the proportion of women than in the general COPD population. Additionally, our analyses were cross-sectional, thus we were not able to demonstrate a causal relationship between low health literacy and disease beliefs. Moreover, we did not evaluate the association of health literacy to adherence through health beliefs. However, we are planning to examine a complete model once we complete our enrollment and follow-up of these participants.

Despite these limitations, our findings have the potential to significantly improve the care of patients with COPD. First, our results add to the literature strongly supporting the need to address the health literacy of patients with COPD, and suggest that self-management education materials for this population should be designed with features that support understanding of printed materials by low-literacy patients, such as teach-to-goal, multi-modal communication (e.g. verbal and print), and optimized formatting of print materials (e.g., low reading grade level, single idea per line, concrete instructions) [43]. Second, they suggest that physicians and other health care providers should consider incorporating questions regarding illness perceptions into their discussions with low health literate patients. By targeting some of the specific beliefs we identified as associated with low health literacy, providers will have the opportunity to counsel patients about their disease, and redress beliefs that have the potential to influence critical self-management behaviors. Given the high risk of poor outcomes and the multiple barriers to self-care, the management of low health literate patients stands to benefit from an integrated approach involving care coordination and care coaching, where specific beliefs could be intensively addressed in a one-to-one fashion. Future research will be needed to assess the best modes of intervening on erroneous health beliefs and their impact on patient health.
